# Possible role of intravenous administration of mesenchymal stem cells to alleviate interstitial cystitis/bladder pain syndrome in a Toll-like receptor-7 agonist-induced experimental animal model in rat

**DOI:** 10.1186/s12894-021-00923-3

**Published:** 2021-11-13

**Authors:** Hidetoshi Tabata, Masanori Sasaki, Yuko Kataoka-Sasaki, Nobuo Shinkai, Koji Ichihara, Naoya Masumori, Jeffery D. Kocsis, Osamu Honmou

**Affiliations:** 1 Department of Urology, Sapporo Medical University School of Medicine, Sapporo, 060-8556 Japan; 2 Department of Neural Regenerative Medicine, Research Institute for Frontier Medicine, Sapporo Medical University School of Medicine, Sapporo, Hokkaido 060-8556 Japan; 3 Department of Neurology, Yale University School of Medicine, New Haven, CT 06510 USA; 4 Center for Neuroscience and Regeneration Research, VA Connecticut Healthcare System, West Haven, CT 06516 USA

**Keywords:** Intravenous, Mesenchymal stem cell, Hunner-type interstitial cystitis

## Abstract

**Background:**

Interstitial cystitis/bladder pain syndrome (IC/BPS) categorized with and without Hunner lesions is a condition that displays chronic pelvic pain related to the bladder with no efficacious treatment options. There are strong associations suggested between Hunner-type IC and autoimmune diseases. Recently, we established an animal model of Hunner-type IC using a Toll-like receptor-7 (TLR7) agonist. Intravenous infusion of mesenchymal stem cells (MSCs) can be used to treat injury via multimodal and orchestrated therapeutic mechanisms including anti-inflammatory effects. Here, we investigated whether infused MSCs elicit therapeutic efficacy associated with the TLR7-related anti-inflammatory pathway in our Hunner-type IC model.

**Methods:**

Voiding behaviors were monitored 24 h prior to the Loxoribine (LX), which is a TLR7 agonist instillation in order to establish a Hunner-type IC model (from − 24 to 0 h) in female Sprague–Dawley rats. LX was instilled transurethrally into the bladder. At 0 h, the initial freezing behavior test confirmed that no freezing behavior was observed in any of the animals. The LX-instilled animals were randomized. Randomized LX-instilled rats were intravenously infused with MSCs or with vehicle through the right external jugular vein. Sampling tissue for green fluorescent protein (GFP)-positive MSCs were carried out at 48 h. Second voiding behavior tests were monitored from 72 to 96 h. After the final evaluation of the freezing behavior test at 96 h after LX instillation (72 h after MSC or vehicle infusion), histological evaluation with H&E staining and quantitative real-time polymerase chain reaction (RT-PCR) to analyze the mRNA expression levels of inflammatory cytokines were performed.

**Results:**

Freezing behavior was reduced in the MSC group, and voiding behavior in the MSC group did not deteriorate. Hematoxylin–eosin staining showed that mucosal edema, leukocyte infiltration, and hemorrhage were suppressed in the MSC group. The relative expression of interferon-β mRNA in the bladder of the MSC group was inhibited. Numerous GFP-positive MSCs were distributed mainly in the submucosal and mucosal layers of the inflammatory bladder wall.

**Conclusion:**

Intravenous infusion of MSCs may have therapeutic efficacy in a LX-instilled Hunner-type IC rat model via a TLR7-related anti-inflammatory pathway.

## Background

Interstitial cystitis/bladder pain syndrome (IC/BPS) is a condition that causes chronic pelvic pain related to the bladder with no efficacious treatment options [1]. The complete picture of IC/BPS remains unclear; however, IC/BPS can be categorized into IC/BPS with and without Hunner lesions [2]. Hunner-type IC is characterized by the cystoscopic finding known as Hunner lesions, reddish mucosal lesions accompanied by abnormal capillary structures. Hunner-type IC has a proven bladder histology that manifests epithelial denudation and chronic inflammatory changes, such as lymphoplasmacytic and mast cell infiltration, stromal fibrosis, and edema [3].

Recently, we established an animal model of Hunner-type IC using a Toll-like receptor 7 (TLR7) agonist [4]. Strong associations occur between Hunner-type IC and autoimmune diseases [5]. Toll-like receptors (TLRs) are key players in the innate immune system and in biological defense mechanisms against external pathogens [6]. Among TLRs, TLR7 is associated with the development and maintenance of inflammation and pain and is considered to contribute to the development of several autoimmune diseases [5]. As we identified an increase in the number of TLR7 immunoreactive cells and in TLR7 mRNA expression in the bladder of Hunner-type IC patients, loxoribine (LX) (a TLR7 agonist) was applied to the bladder of C57BL/6N female mice to induce edema, congestion and inflammation and was associated with significantly increased TLR7-mRNA expression (4). The animals instilled with LX demonstrated increased licking behavior, voiding frequency, and afferent nerve activities associated with increased single-voided volume and intercontraction interval of micturition per cystometry measurements [4].

Cellular therapy for IC/BPS, using mesenchymal stem cells (MSCs) derived from multiple sources, has been reported to provide therapeutic efficacy. The proposed mechanisms by which transplanted MSCs restrain IC/BPS symptoms include regeneration of damaged bladder tissue through the Wnt pathway [7, 8], anti-tissue fibrosis [9], anti-apoptosis via the AKT/mTOR pathway [10], antioxidant [11, 12], and anti-inflammatory effects including mast cell infiltration and inhibition of inflammatory cytokines [13, 14]. However, specific pathways inhibiting inflammation in MSCs have not been identified.

Here, we investigated whether intravenous infusion of MSCs elicits therapeutic efficacy to reduce inflammation in our established Hunner-type IC model with a TLR7 agonist. Bladder pain-like behavior and voiding behaviors were monitored. In addition, histopathological gene expression analyses was carried out to identify a TLR7-related anti-inflammatory pathway, with the intent of utilizing these data to develop approaches to reduce the symptoms in this experimental Hunner-type IC model.

## Materials and methods

### Preparation of MSCs from bone marrow

MSCs were cultured as described in our previous studies [15, 16]. Briefly, the bone marrow was collected from the femoral bones of adult wild-type Sprague–Dawley (SD) rats and GFP-expressing rats [W-Tg (CAG-GFP)184Ys], diluted to 25 ml with Dulbecco's modified Eagle's medium (DMEM) (Sigma, St. Louis, MO, USA), supplemented with 10% heat-inactivated fetal bovine serum (FBS) (Thermo Fisher Scientific Inc., Waltham, MA, USA), 2 mM l-glutamine (Sigma), 100 U/ml penicillin, and 0.1 mg/ml streptomycin (Thermo Fisher Scientific Inc.), and incubated for 3 days (5% CO_2_, 37 °C). When the cultures almost reached confluence (over 95%), adherent cells were detached using trypsin–EDTA solution (Sigma) and subcultured on a 150 mm^2^ tissue culture dish (1030–150: IWAKI, Tokyo, Japan; surface area: 148 cm^2^) at 5 × 10^5^ cells/ml in 14 ml culture medium; thus, the plating density was approximately 3.4 × 10^3^/cm^2^. The surface antigen phenotype of MSCs was as follows: CD45^−^, CD73^+^, CD90^+^, and CD106^−^ [17, 18]. The cultured MSCs were used for infusion after three passages. For infusion, the supernatants discarded and MSCs (1.0 × 10^6^ cells) were resuspended in fresh 1 ml DMEM and injected.

### Hunner-type IC model

Sixty-two female SD rats, aged 8 weeks, were used. The animals were housed under standard laboratory conditions with a 12:12 h light: dark cycle and free access to food and water. Under anesthesia with ketamine and xylazine (75 and 10 mg/kg, respectively; i.p.), a polyethylene catheter (PE-50) was atraumatically inserted into the bladder of rats, transurethrally. After urine was drained, 200 µl of 4.5 mM Loxoribine (LX) (AdipoGen Life Sciences, Liestal, Switzerland) (n = 36), a selective TLR7agonist [or distilled water (DW) for sham (n = 14)], was instilled slowly. The catheter was kept for 60 min, after which the instilled liquid was drained, and the animals were allowed to recover from anesthesia.

### Experimental protocol

**Fig. 1:**
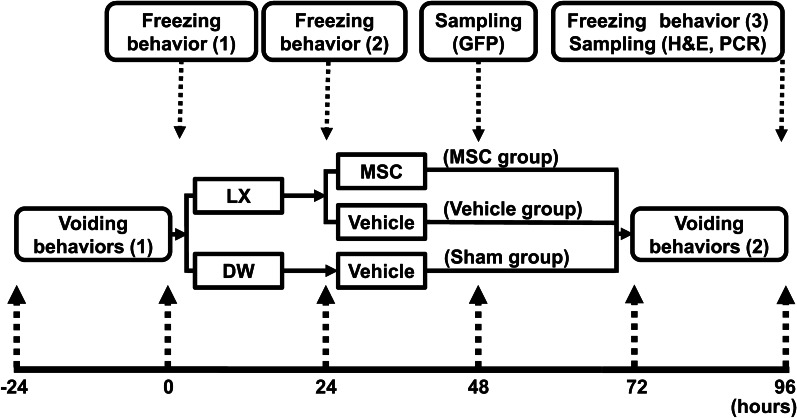
Experimental protocol

**Fig. 2:**
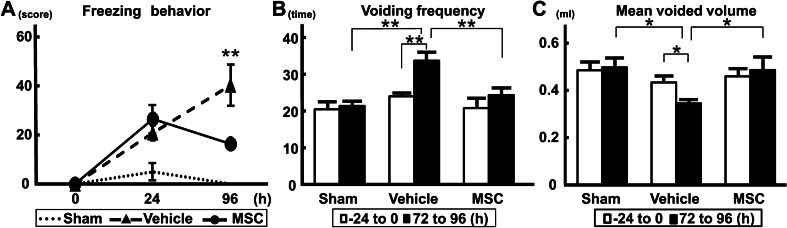
Urological evaluations. **a** Bladder pain-like behavior; **b** voiding frequency; **c** mean voided volume. n = 6/group. ANOVA with the Tukey–Kramer post hoc test. *p < 0.05, **p < 0.01. *h* “hour”

The experimental protocol is illustrated in Fig. 1. Voiding behaviors (1) were monitored 24 h prior to LX or DW instillation, to establish a Hunner-type IC model (from − 24 to 0 h). At 0 h, the initial freezing behavior (1) test confirmed that no freezing behavior was observed in any of the animals (Fig. 2a). LX was instilled into 48 rats, and DW was instilled into 14 rats. After the second freezing behavior (2) test following LX or DW instillation at 24 h, the 12 LX-instilled rats that displayed no freezing behavior were excluded. Thus, we included 36 LX-instilled rats and 14 DW-instilled rats for this study. Eight of the 36 LX-instilled rats were used for the detection of GFP-expressing MSCs (GFP-MSCs). GFP-MSCs were infused into LX-instilled animals (n = 4), and MSCs derived from wild-type animals were infused into LX-instilled animals (n = 4), at 48 h (24 h after MSC or vehicle infusion). The remaining LX-instilled animals (n = 28) were randomized. Randomized LX-instilled rats were intravenously infused with MSCs (MSC group: 1.0 × 10^6^ cells in 1 ml fresh DMEM) or with vehicle (Vehicle group: 1 ml fresh DMEM), and the DW-instilled rats were infused with vehicle only (Sham group) through the right external jugular vein [15]. Second voiding behavior [2] tests were monitored from 72 to 96 h. After the final evaluation of the freezing behavior [3] test at 96 h after LX instillation (72 h after MSC or vehicle infusion), histologic evaluations and quantitative real-time polymerase chain reaction (RT-PCR) were performed.

### Transplantation procedure

The rats used in this study were anesthetized with an intraperitoneal (IP) injection of ketamine and xylazine (90/4 mg/kg) and received intravenous infusion of MSCs (1.0 × 10^6^) in 1.0 ml total fluid volume (DMEM) or vehicle (1.0 ml fresh DMEM alone) via right external jugular vein. All rats were injected subcutaneously daily with cyclosporine A (10 mg/kg).

### Freezing behavior

Freezing behavior was defined as bladder pain-like behavior of rats, indicated by cessation of movement and pointing of the nose toward the lower abdomen without licking. If the freezing behavior occurred within a 5-s interval, it was scored as one positive event. The number of positive events of the respective behaviors was counted for 30 min [19].

### Voiding behavior

Voiding behaviors (voiding frequency and measurement of voided volume) were recorded for 24 h using a metabolic cage (Urination Monitor-100, Melquest Ltd., Toyama, Japan), after 24 h of adaptation to the environment [4]. The voiding frequency was recorded for 24 h; after a 5-min interval, it was defined as the next voiding. The cutoff for voided volume in this experiment was defined as 0.03 ml. These settings were applied according to the findings from our pilot study. During recording, the rats were allowed free access to water and food.

### Histopathological analyses

Rats were deeply anesthetized with an intraperitoneal injection of ketamine (50 mg/kg) and xylazine (10 mg/kg), perfused with phosphate-buffered saline (PBS) and 4% paraformaldehyde (PFA) in 0.1 M phosphate-buffer (PB), 96 h after LX instillation (72 h after MSC or vehicle infusion). Then, the bladders were isolated from each group and opened at the dorsal side of the bladder neck, along the midline towards the dome. After macroscopic evaluation, bladders were placed in 4% formaldehyde and embedded in paraffin for microscopic evaluation via hematoxylin–eosin (H&E) staining. For H&E staining, paraffin-embedded sections were cut (3 μm) using a Leica RM2265 microtome (Leica Microsystems, Wetzlar, Hesse-Darmstadt, Germany). The deparaffinized sections were stained with hematoxylin for 5 min at room temperature, followed by staining with eosin solution (1% eosin + 95% ethanol + acetate) for 3 min at room temperature and then photographed (BZ-9000; Keyence, Osaka, Japan). During histological evaluation, ten fields of each slide were scored for mucosal edema, leukocyte infiltration, hemorrhage, and mucosal abrasion [20]. Edema was evaluated at × 200 magnification. No edema was scored as 0, minimal edema (no change in connective tissue thickness) as 1, moderate edema (connective tissue thickness increased by < twofold) as 2, and severe edema (connective tissue thickness increased by > twofold) as 3. Leukocyte infiltration was evaluated in the mucosa at × 400 magnification. No extravascular leukocytes were scored as 0, > 20 leukocytes as 1, 20–45 leukocytes as 2, and > 45 leukocytes as 3. Mucosal hemorrhage and mucosal abrasion were evaluated at × 100 magnification. The presence of hemorrhage and mucosal abrasion was scored as 1, and no change was scored as 0. The total score of all fields of view for mucosal edema, leukocyte infiltration, mucosal hemorrhage, and mucosal abrasion was divided by the maximum possible score and then multiplied by 100 [20].

### RT-PCR

Quantitative RT-PCR was performed as previously described [15]. After 96 h of LX instillation (72 h after MSC or vehicle infusion), the animals were anesthetized with an intraperitoneal injection of ketamine (50 mg/kg) and xylazine (10 mg/kg), and bladders from each group were collected using a dissecting microscope. Total RNA was extracted using an RNeasy Plus Mini kit (#74134, Qiagen, Valencia, CA, USA) according to the manufacturer’s instructions. The RNA concentration was quantified by determining the optical density at 260 nm. RNA (1 μg) was reverse transcribed into cDNA using SuperScript III reverse transcriptase (Qiagen) and oligo-dT. Real-time PCR for each sample was performed in triplicate with TaqMan Universal Master Mix II with no UNG (Thermo Fisher Scientific Inc.). The following sets of specific primers and TaqMan probes were purchased from Thermo Fisher Scientific Inc.: glyceraldehyde-3-phosphate dehydrogenase (GAPDH) (TaqMan rodent GAPDH control reagents, Rn01775763-g1) as an endogenous control, and TLR7 (Rn01771083) and interferon (*IFN*)-β (Rn00569434) as target genes. The reactions were run on an ABI-StepOne real-time PCR system (Thermo Fisher Scientific Inc.) using a 48-well plate format. The cycling conditions included an initial denaturation phase at 95 °C for 3 min, followed by 40 cycles of 95 °C for 15 s and 55 °C for 60 s. Relative quantification of target gene expression was performed using the comparative threshold cycle method, according to the manufacturer’s guidelines.

### Detection of GFP expressing MSCs

Detection of GFP expressing MSCs was performed as previously described [15]. One day after injection of GFP-MSCs from GFP-expressing rats and non-GFP-MSCs from wild-type SD rats, transcardial perfusion (4% PFA) under anesthesia with an intraperitoneal injection of ketamine (50 mg/kg) and xylazine (10 mg/kg) was performed to dissect out the bladder. After dissection, the bladders were fixed in 4% PFA for 1 h at 4 °C. Then, the frozen embedded bladder was cut into 12-μm thick sections with a cryostat and mounted on glass slides. Slides were washed thrice in PBS with Tween 20 (0.1%) (PBST) and blocked in normal goat serum (10%) and Triton X (0.3%) in PBS at room temperature for 30 min. The sections were then incubated overnight at 4 °C with the primary antibody (chicken anti-GFP antibody, 1:1000; ab13970, Abcam, Cambridge, MA, USA), diluted with normal goat serum (5%), Triton X (0.3%), and PBS. After four washes in PBST, the sections were incubated with the secondary antibody (1:2000; AF 488-conjugated goat anti-chicken immunoglobulin Y, Abcam, 150,173) for 1 h and counterstained with DAPI. The sections were then examined using a confocal microscope (Ex/Em, 405/488: LSM780 ELYRA system; Carl Zeiss, Oberkochen, Germany).

### Statistical analysis

All statistical analyses were performed using SPSS 18 (SPSS Inc., Chicago, IL, USA). Comparisons were performed using one-way analysis of variance with the Tukey–Kramer post hoc test.

## Results

### The freezing behavior score was decreased following MSC infusion

There were no significant differences among the three experimental groups (MSC, vehicle, and sham) in the initial freezing behavior (1) test at 0 h. At 24 h after LX or DW instillation (prior to MSC or vehicle infusion), the scores of the second freezing behavior (2) test were increased in the LX-instilled groups (both vehicle and MSC groups). At 96 h, the freezing behavior score was further increased in the vehicle group; however, the score in the MSC group decreased (*p* < 0.01) (Fig. 2a).

### The intravenous infusion of MSCs suppressed pelvic pain evaluated with voiding behaviors

There were no significant differences among the three groups in terms of voiding frequency (Fig. 2b) and mean voided volume (Fig. 2c) from − 24 to 0 h (*p* = 0.55). It was difficult to measure stable voiding behaviors prior to MSC or vehicle infusion, possibly because of the effects of anesthesia and intravesical activity of catheter implantation for instillation of LX into the bladder. In addition, a 24 h period is required to measure voiding behaviors (see Materials and Methods). The voiding frequency in the vehicle group at 96 h (from 72 to 96 h) was elevated compared with that in the MSC and sham groups (*p* < 0.01); there was no difference between the sham and MSC-infused groups. The voiding frequency at 96 h was also higher than that at 0 h (from − 24 to 0 h) in the vehicle group, although there were no differences between the sham and MSC-infused groups. The mean voided volume in the vehicle group at 96 h (from 72 to 96 h) was lower than that in the MSC and sham groups (*p* < 0.05), and there was no difference between the sham and MSC-infused groups. The mean voided volume at 96 h was lower than that at 0 h (from − 24 to 0 h) in the vehicle group, although there was no difference between the sham and MSC-infused groups. These results suggest that intravenous infusion of MSCs suppressed pelvic pain following LX instillation in the bladder, indicating the therapeutic efficacy of infused MSCs for experimental IC.

### Intravenous infusion of MSCs inhibited histopathological damage

**Fig. 3:**
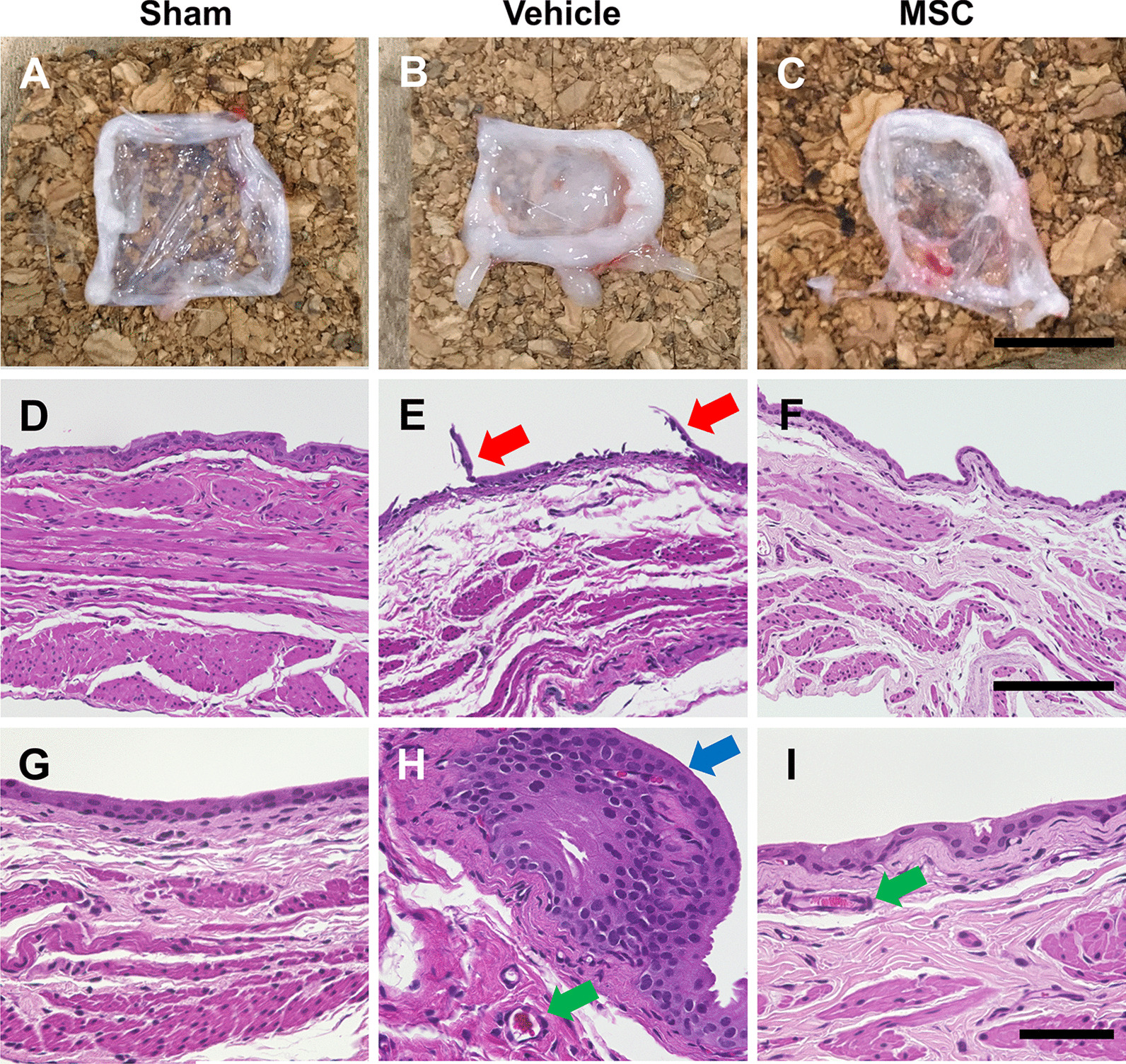
Representative macroscopic and histological images of bladder at 96 h following intravenous infusion of MSCs or vehicle in a LX-instilled rat model of Hunner-type IC. (**a, d, g**) Sham, (**b, e, h**) vehicle, and (**c, f, i**) MSC groups. Red arrows indicate mucosal ablation. Blue arrows indicate leukocyte infiltration. Green arrows indicate hemorrhage. Scale bars = 1 cm (**a–c**), 150 μm (**d–f**), and 50 μm (**g–i**)

**Fig. 4:**
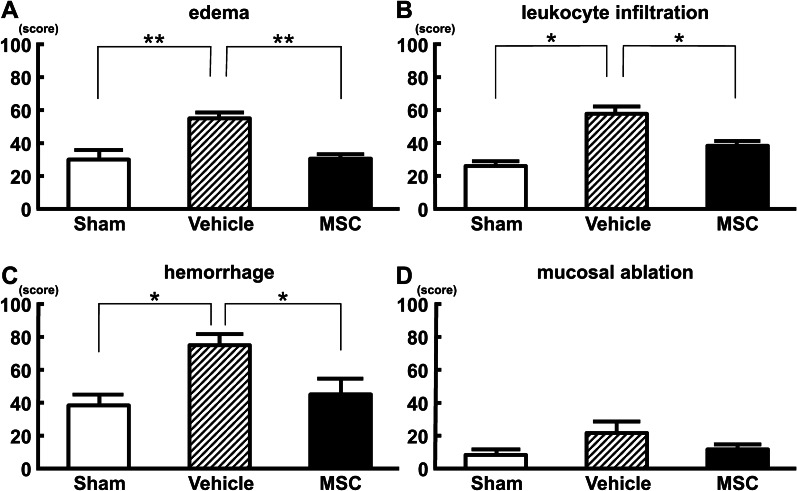
Histological evaluation. **a** Edema, **b** leukocyte infiltration, **c** hemorrhage, and **d** mucosal ablation. n = 6/group. ANOVA with the Tukey–Kramer post hoc test. *p < 0.05, **p < 0.01

Macroscopic observation revealed that the bladder wall in the vehicle group (Fig. 3b) was thicker than that in the sham group (Fig. 3a), and the bladder wall in the MSC group (Fig. 3c) was thinner than that in the vehicle group. Histopathological evaluation using H&E staining in the vehicle group revealed mucosal edema (Fig. 3e), mucosal ablation (Fig. 3e, red arrows), hemorrhage (Fig. 3h, green arrow), and leukocyte infiltration (Fig. 3h, blue arrows), although H&E staining in the sham group (Fig. 3d, g) showed no thickening and edema in the mucosal and submucosal layers, as well as no inflammatory features such as hemorrhage or leukocyte infiltration. In the vehicle group, these inflammatory features were less observed compared to the MSC group (Fig. 3f, i). Mucosal edema (Fig. 4a, *p* < 0.01), leukocyte infiltration (Fig. 4b: *p* < 0.05), and hemorrhage (Fig. 4c, *p* < 0.05) were evident in the vehicle group after LX instillation compared with the sham group. These changes were suppressed in the MSC-treated group. Although a trend was observed, there were no significant differences among the three groups in mucosal ablation (Fig. 4d, *p* = 0.16). These results indicate that intravenous infusion of MSCs inhibited histopathological damage following LX instillation in the bladder.

### Infused MSCs reduced the expression of IFN-β in the TLR7 expressed bladder wall following LX instillation

**Fig. 5:**
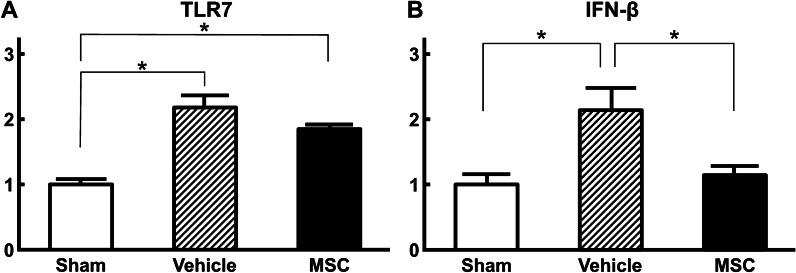
mRNA expression of TLR7 (**a**) and IFN-β (**b**) in the bladder wall at 96 h following infusion of MSCs or vehicle in a LX-instilled rat model of Hunner-type IC. n = 8/group. ANOVA with the Tukey–Kramer post hoc test. *p < 0.05.

The relative expression of TLR7-mRNA in the bladder wall in both vehicle and MSC groups was elevated following LX instillation compared with the sham group (Fig. 5a: *p* < 0.01), and there was no significant difference between the MSC and vehicle groups. The relative expression of *IFN-β* mRNA in the bladder wall in the vehicle group was elevated following LX instillation compared with that in the MSC and sham groups (Fig. 5b, *p* < 0.05). Even though TLR7 levels were elevated in the MSC group after LX instillation, *IFN-β* mRNA levels were not.

### Accumulated MSCs in the inflammatory bladder wall

**Fig. 6:**
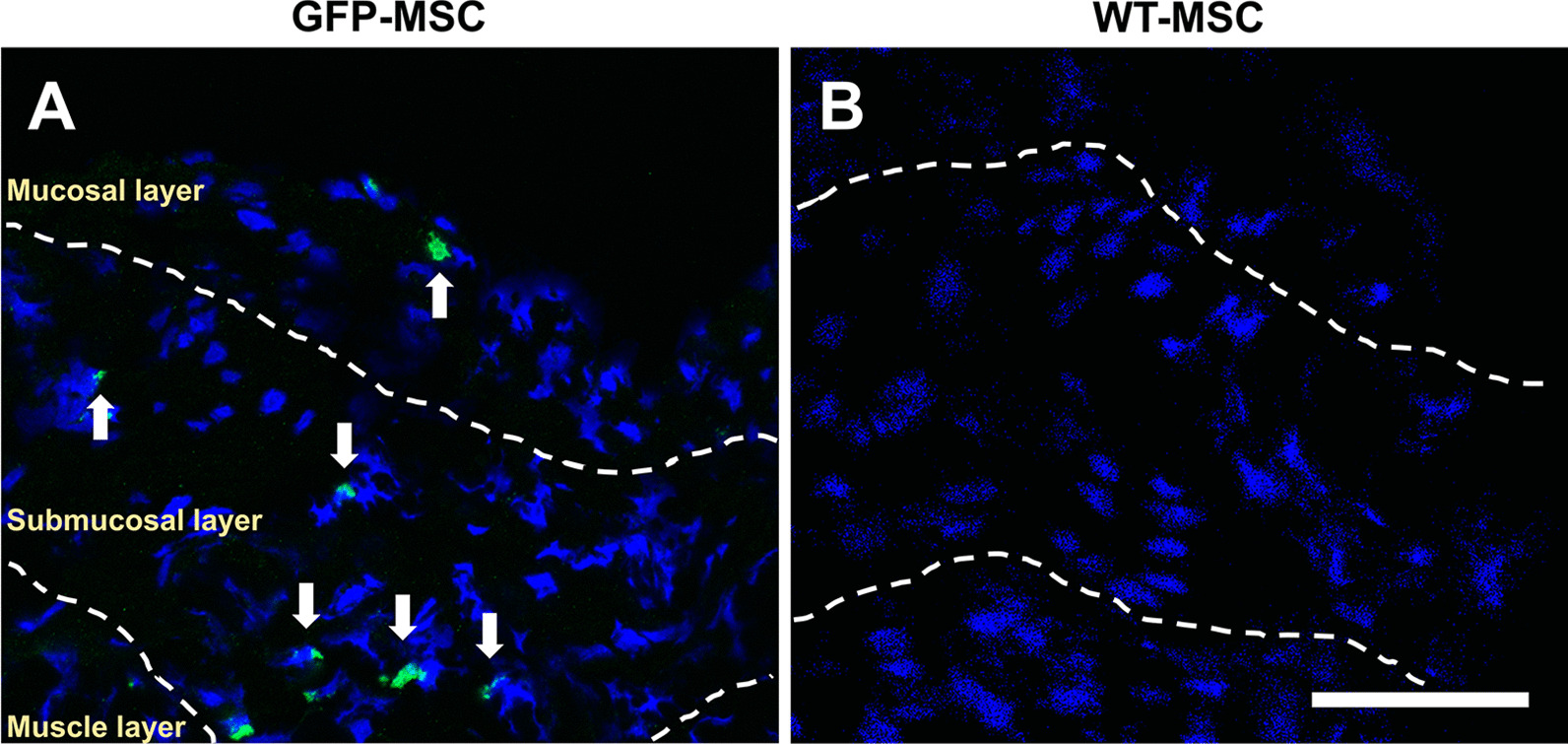
Distribution of GFP-MSCs in the bladder wall at 48 h following infusion of MSCs or vehicle in a LX-instilled rat model of Hunner-type IC. **a** GFP-MSCs; **b** MSCs derived from wild type rats. Dashed lines indicate the borders of mucosal, submucosal and muscle layers. Arrows indicate GFP-MSCs. Scale bar = 50 μm

GFP-MSCs were identified in the inflammatory bladder wall via observation of green fluorescence (Fig. 6a). Numerous infused GFP-MSCs were more extensively distributed in the submucosal and mucosal layers and few GFP-MSCs were present in the muscle layer (Fig. 6a). The accumulation of the infused GFP-MSCs demonstrated a homing effect in the LX-instilled bladder, suggesting that GFP-MSCs might accumulate at the inflammation lesion site in the bladder, where we observed leukocyte infiltration in the vehicle group (Fig. 3h). To confirm whether the infused MSCs showed autofluorescence at the wavelengths used to study GFP fluorescence, we examined sections of samples from animals infused with non-GFP-MSCs derived from wild-type SD rats. No GFP^+^ cells were observed in these rats (Fig. 6b).

## Discussion

We found that intravenous infusion of MSCs reduced the symptoms of IC/BPS in our newly established animal model of Hunner-type IC using a TLR7 agonist. The IC/BPS related freezing behavior was improved (reduced), and the voiding behaviors scored as voiding frequency and mean voided volume did not deteriorate following infusion of MSCs compared with the vehicle group. Macroscopic observation and histological analysis using H&E staining demonstrated that preservation of the bladder wall structure was clearly observed in the MSC group. The bladder wall thickness in the MSC group was thinner than that in the vehicle group, and several pathological features, including hemorrhage, edema, and leukocyte infiltration, showed improvement in bladder wall pathology. We observed a significantly higher expression of TLR7 mRNA in the bladder wall in our IC/BPS model. However, the expression of *IFN-β* mRNA in the MSC group was significantly reduced compared to that in the vehicle group. Accumulated GFP-MSCs were found mainly in the submucosal and mucosal layers in the inflammatory bladder wall after intravenous infusion of GFP-MSCs. These data suggest that MSCs might contribute to the downregulation of *IFN-β* and thus reduction in inflammation.

**Fig. 7:**
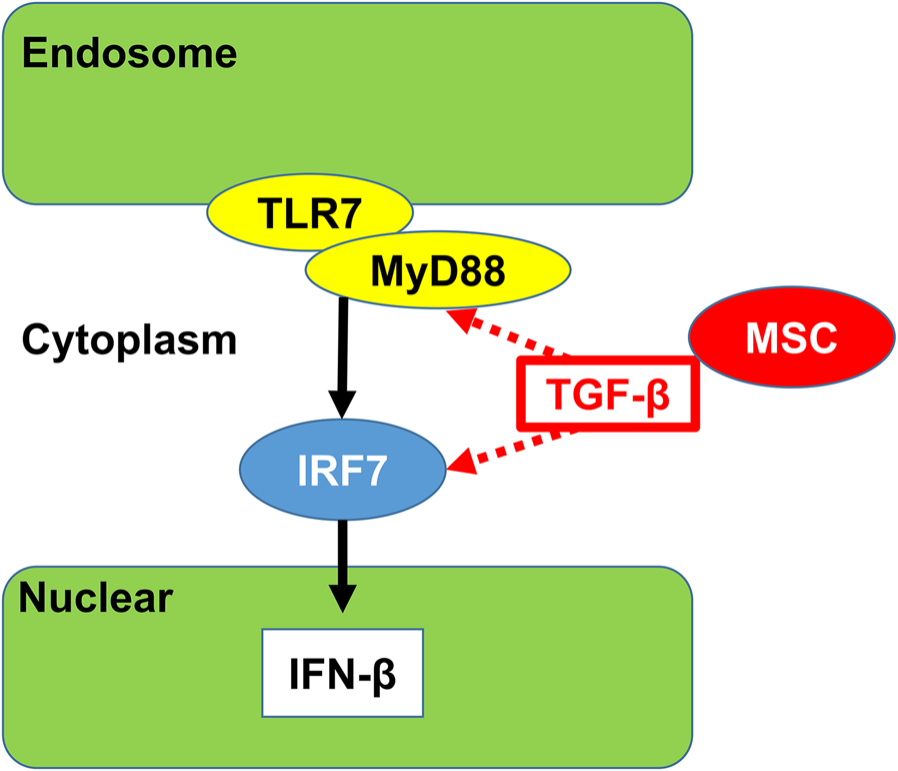
Possible therapeutic mechanism of infused MSCs. TLR7: Toll like receptor 7, MyD88: myeloid differentiation primary response 88, TGF-β: transforming growth factor beta, IRF7: interferon regulatory factor 7, IFN-β: interferon beta

Although the etiology and pathophysiology of IC/BPS are uncertain, autoimmune disorders are common comorbidities of IC/BPS [21]. Thus, autoimmunity could be among the possible causes of IC/BPS [22]. In the animal model of Hunner-type IC induced by a TLR7 agonist used here [4], we elucidated that intravenous infusion of MSCs provides therapeutic efficacy for the Hunner-type IC model by downregulating the expression of *IFN-β* in a TLR7-induced rat model. Previous studies indicated that TLR7-myeloid differentiation primary response 88 (MyD88) signaling, which activates interferon regulatory factor 7 (IRF-7), results in robust induction of *IFN-β* [23, 24]. It has been reported that MSCs secrete transforming growth factor beta *(TGF-β)* [25] and they can stimulate macrophages to secrete *TGF-β* in sites of inflammation [26]. Infused MSCs accumulated in the mucosal and submucosal layers of the bladder wall and might contribute to increased levels of *TGF-β* [25]. *TGF-β* inhibits the phosphorylation of IRF-7, reduces the protein level of MyD88 [27], and downregulates the production of *IFN-β* [28]. The expression level of TLR7 was not significantly reduced in our model system, although *IFN-β* was downregulated following intravenous infusion of MSCs. Therefore, it is conceivable that infused MSCs might play an important role in reducing the expression of *IFN-β* (Fig. 7), thereby promoting the reduction of inflammation in the bladder wall. Thus, a novel finding of this study is that the intravenous infusion of MSCs has therapeutic effects on Hunner-type IC by downregulating the expression of IFN-β in this TLR7-induced Hunner-type IC model. Therefore, using this unique model, a novel pathway for inhibiting the inflammation induced by infused MSCs has been proposed.

It is important to contextualize that Ichihara et al. used mice to develop this Hunner-type IC model with TLR7; however, we used rats because most cellular transplantation studies with Hunner-type IC models have used rats [7–10, 13, 14, 29]. We used the same concentration of LX as that in the mouse model (4.5 mM, 100 μm) [4], but we increased the volume of LX (4.5 mM, 200 μm) in the current study after considering the bladder volume of rats [30]. A potential strength of this study is the use of a TLR7-induced Hunner-type IC rat model system compared with other commonly used IC/BPS model systems induced with cyclophosphamide [31, 32], lipopolysaccharide [33], hydrochloric acid (HCl) [7, 13], uroplakin [14, 29] and ketamine [9].

We propose that the TLR7-related anti-inflammatory pathway is associated with restraining IC/BPS pathology. This study provides additional insights into the anti-inflammatory mechanism of infused MSCs, in addition to the infiltration of mast cells and reduction of inflammatory cytokines [13, 14].

A limitation of this study is that as mentioned above, that we employed the methods used in the original paper by Ichihara et al. [4]. The study period by Ichihara et al. was 96 h which we used in this study. We also would like to stress that our preliminary study with our established Hunner-type IC model with TLR7 agonist demonstrated that the bladder pain-like behavior and voiding behaviors did not last more than a week. Thus, we focused on acute therapeutic effects of infused MSCs in this study. We also realize that elucidation of the precise molecular mechanism underlying the MSC therapy is important and future studies should be performed to establish a more appropriate model for longer term study and study of corresponding pathways and proteins using western blot and ELISA.

## Conclusions

Intravenous infusion of MSCs may have an impact on the mitigation of IC/BPS symptoms via *IFN-β* downregulation in a TLR7-induced rat model of Hunner-type IC model system.

## Data Availability

The datasets used and/or analyzed during the current study are available from the corresponding author on reasonable request.
